# Perceived Treatment Satisfaction and Effectiveness Facilitators Among Patients With Chronic Health Conditions: A Self-Reported Survey

**DOI:** 10.2196/13029

**Published:** 2020-03-06

**Authors:** Kathryn Volpicelli Leonard, Courtney Robertson, Amrita Bhowmick, Leslie Beth Herbert

**Affiliations:** 1 Health Union LLC Philadelphia, PA United States; 2 Department of Health Behavior, Gillings School of Global Public Health University of North Carolina Chapel Hill, NC United States

**Keywords:** treatment effectiveness, patient satisfaction, migraine, multiple sclerosis, rheumatoid arthritis

## Abstract

**Background:**

Approximately 50% of patients are nonadherent to prescribed medications. Patient perception regarding medication effectiveness has been linked to improved adherence. However, how patients perceive effectiveness is poorly understood.

**Objective:**

The aim of this study was to elucidate factors associated with perceived treatment satisfaction and effectiveness among patients with chronic health conditions.

**Methods:**

We conducted a descriptive study using a cross-sectional survey design. We administered a Web-based survey to participants with migraine, multiple sclerosis (MS), or rheumatoid arthritis (RA). Patients were recruited from established online communities of Health Union. Descriptive statistics, correlations, and comparison tests were used to examine outcomes.

**Results:**

Data were collected from 1820 patients: 567 with migraine, 717 with MS, and 536 with RA. The majority of participants were female (1644/1820, 90.33%), >40 years old (1462/1820, 80.33%), and diagnosed >5 years ago (1189/1820, 65.33%). Treatment satisfaction and perceived medication effectiveness were highly correlated (*r*=0.90, *P*<.01). Overall, three temporal factors were positively correlated with satisfaction or perceived effectiveness: time on current medication (satisfaction *r_s_*=0.22, *P*<.01; effectiveness *r*_s_=0.25, *P*<.01), time since diagnosis (satisfaction *r*_s_=0.07, *P*<.01; effectiveness *r*_s_=0.09, *P*<.01), and time on treatment (effectiveness *r*_s_=0.08, *P*<.01).

**Conclusions:**

Findings validated the strong relationship between treatment satisfaction and perceived effectiveness. Understanding the (1) positive relationship between time and treatment satisfaction and effectiveness and (2) factors associated with determining medication effectiveness can help clinicians better understand the mindset of patients regarding treatment. Clinicians may be better prepared to elicit patient beliefs, which influence medication adherence, for people diagnosed with chronic health conditions.

## Introduction

### Background

The World Health Organization reported that in developed countries, approximately 50% of patients with chronic health conditions do not adhere to the medication they have been prescribed [1]. Adherence and compliance to treatment are important in any disease context but can be particularly challenging in the context of chronic health conditions that require sustained adherence, even in the absence of acute symptoms and with regimens that can be logistically and economically challenging [1,2]. In turn, low levels of adherence and compliance can have a dramatic detrimental impact on symptomology, overall disease course, and health care costs [1-3]. For patients with chronic conditions, noncompliance can mean reduced quality of life and swifter disease progression; for the health care system as a whole, nonadherence increases the societal cost burden (eg, for avoidable health care professional [HCP] visits and hospitalizations) [3-7]. Adherence and compliance to treatment are, therefore, widely researched topics, with a complex set of predictors summarized by Jin et al [5] into categories related to patient-determined factors (demographics, beliefs, motivations, etc), treatment logistics, social and economic factors, health care availability and accessibility, and disease experiences.

Several studies have looked closely at one particular component of that matrix: patient beliefs about therapy. For example, Rajpura and Nayak [8] reported that positive beliefs regarding medication predicted adherence to medication among elders with hypertension. Patient satisfaction with treatment (in addition to the more general construct of patient satisfaction with care) has also been associated with better adherence and compliance, including research done among patients seeking chronic pain treatment, patients with type 2 diabetes, patients with chronic obstructive pulmonary disease, patients with cystic fibrosis, patients with depression, and patients with hypertension [9-14].

In addition, research has supported a role for treatment efficacy perceptions in predicting better adherence and compliance. Bender and Bender [15] found that, among patients with asthma, concerns about diminishing treatment effectiveness over time played a secondary role in adherence behaviors, behind more frequently mentioned factors such as safety, cost, and perceived disease severity. Although not treatment efficacy *per se*, Horne and Weinman [16] also found that patients who believed that their prescribed medication was necessary for maintaining health also reported higher compliance.

Research has also shown a strong connection between treatment satisfaction and perceived treatment effectiveness. For example, the Treatment Satisfaction Questionnaire for Medication (TSQM) incorporates an element of perceived treatment effectiveness as 1 of the 4 domains in determining treatment satisfaction. Specifically, the TSQM includes questions related to the impact of the medication on disease and symptoms that, along with all other dimensions, have been shown to be highly reliable and valid constructs. Relevant to the current research questions, the TSQM was also originally validated among patients with chronic conditions (arthritis, asthma, depression, type 1 diabetes, hypercholesterolemia, hypertension, migraine, and psoriasis) [17], and was found to be a useful tool for measuring treatment satisfaction among multiple sclerosis (MS) patients [18].

### Objectives

In summary, several studies have demonstrated a relationship between treatment satisfaction and adherence and compliance, as well as between perceived treatment effectiveness and adherence and compliance. Moreover, a strong link has been established between treatment satisfaction and perceived treatment effectiveness.

However, although some research has demonstrated the importance patients place on efficacy over tolerability and ease of administration [19], less research has focused on the basis of patient perceptions of treatment effectiveness.

**Figure 1:**
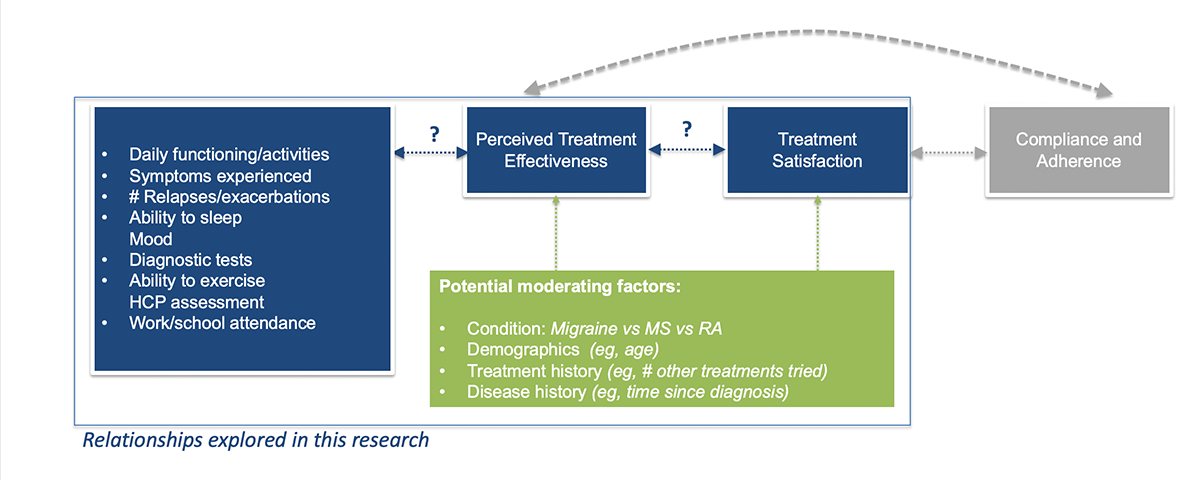
Relationships explored in research on how patients determine if a treatment is working for them. HCP: health care practitioner; MS: multiple sclerosis; RA: rheumatoid arthritis.

The aim of this study was, therefore, to better understand the factors that patients rely on when making personal evaluations of treatment effectiveness, including the role of quality of life improvements, symptoms, and HCP assessment. Secondarily, the research further explored the relationship between perceived treatment effectiveness and treatment satisfaction among patients with chronic conditions (Figure 1). The analyses focused on patients with 1 of 3 chronic conditions (migraine, MS, or rheumatoid arthritis [RA]).

## Methods

### Recruitment

We conducted a descriptive study using a cross-sectional survey design. A Web-based survey was administered from June 17, 2017, to July 30, 2017, among 3 online communities of Health Union (Philadelphia, PA, The United States). Potential participants were recruited as a convenience sample. We employed this common nonprobability sampling technique given its efficiency, ease of implementation, low cost, and the exploratory nature of the questions posed herein.

Links to the survey were posted on the 3 Health Union community sites and associated Facebook pages: Migraine.com, MultipleSclerosis.net, and RheumatoidArthritis.net. We chose to survey these specific patient communities as these patients are apt to have chronic conditions that often require >1 medication to treat, as well as the potential for patients to need to try several medications before finding the one that works for them. Participants were eligible to participate in the survey if they were at least 18 years old, lived in the United States, and previously received a diagnosis from an HCP of the respective health condition of the community (ie, migraine, MS, and RA).

The research was conducted in accordance with all applicable regulations as outlined in the Declaration of Helsinki and was exempt from institutional review. Informed consent was obtained from all participants before completing the Web-based survey. Participants were informed about the voluntary nature of the survey, information being collected, anonymous nature of data collection, and the expected time for survey completion. In addition, no identifiers were collected from participants; however, Internet Protocol (IP) addresses of participants were used to ensure that the questionnaire could only be completed once by any individual. Duplicate entries were avoided by preventing users with the same IP address from accessing the survey more than once during the study period.

### Data Collection

Electronic data collection was conducted through SurveyMonkey (SMVK Inc, California), with data protection provided through its security measures. After reviewing information about the nature of the survey, participants completed a survey consisting of up to 30 multiple-choice questions and an optional free-response item. Responses were completely anonymous, and no compensation was provided for participation.

### Measures

The survey included questions on demographics, treatment journey, and treatment satisfaction and effectiveness. Targeted questions were asked based on where participants were in their journey, that is, having never taken medication, taken medication in the past but stopped, and currently taking medication. In addition, questions were asked to gain a deeper understanding of the number of medications tried and what prompted switching medications. Satisfaction was measured on a 7-point scale, ranging from 1=Not at all satisfied to 7=Extremely satisfied. Perceived treatment effectiveness was also measured on a 7-point scale, ranging from 1=Not at all effective to 7=Extremely effective.

After completing the multiple-choice questions, participants were given the option of completing an open-ended response to share additional observations or concerns about treating their condition. Participants were instructed that *medication* referred to both over-the-counter and prescription medications throughout the survey.

### Statistical Analysis

Several of the collected responses to demographic and other types of questions were utilized to stratify participants for further comparison: age (<40, 40-49, 50-59, and >60), time since diagnosis calculated based on current age and age at diagnosis (<2 years, 2-5 years, and >5 years), number of medications ever taken for health condition (< median for condition and > median for condition), and frequency of HCP visits (once a month or more, every 2-3 months, twice a year, and once a year or as needed). Demographic categories were reported using descriptive statistics, and comparisons between categories were evaluated using chi-squared analyses. Differences in mean Likert scores among categories were evaluated using analysis of variance. Correlation between Likert scores and ordinal variables was conducted using the Spearman rank-order correlation, whereas correlation between Likert scores was conducted using the Pearson correlation. Data analysis used *P*<.05 to determine statistical significance.

## Results

The survey was completed by 1820 participants—567, 717, and 536 diagnosed with migraine, MS, and RA, respectively **(**Table 1**)**. For purposes of this research, data of participants currently using a medication for their condition (N=1641) were analyzed (migraine, n=524; MS, n=617; RA, n=500).

**Table 1:** Demographics of survey respondents currently using a medication for their condition.

Demographic factors		Migraine (n=524), n (%)	MS^a^ (n=617), n (%)	RA^b^ (n=500), n (%)	All (N=1641), n (%)
**Age**					
	<40	124 (23.7)	125 (20.3)	81 (16.2)	330 (20.11)
	40-49	159 (30.3)	141 (22.9)	110 (22.0)	410 (24.98)
	50-59	165 (31.5)	233 (37.8)	157 (31.4)	555 (33.82)
	60+	76 (14.5)	118 (19.1)	152 (30.4)	346 (21.08)
**Sex**					
	Female	501 (95.6)	513 (83.1)	478 (95.6)	1492 (90.92)
	Male	23 (4.4)	104 (16.9)	22 (4.4)	149 (9.08)
**Number of medications**					
	< Median^c^	290 (55.3)	356 (57.7)	261 (52.2)	907 (55.27)
	> Median^c^	234 (44.7)	261 (42.3)	239 (47.8)	734 (44.73)
**Frequency of health care professional visits**					
	Once a month or more	117 (22.3)	34 (5.5)	86 (17.2)	237 (14.44)
	Every 2-3 months	229 (43.7)	225 (36.5)	355 (71.0)	809 (49.30)
	Twice a year	74 (14.1)	271 (43.9)	52 (10.4)	397 (24.19)
	Once a year, when I relapse/need to	104 (19.8)	87 (14.1)	7 (1.4)	198 (12.07)

^a^MS: multiple sclerosis.

^b^RA: rheumatoid arthritis.

^c^The median number of medications for patients with migraine was 7, for patients with multiple sclerosis was 2, and for patients with rheumatoid arthritis was 4; across all respondents the median number was 4.

### Medication Satisfaction and Perceived Treatment Effectiveness

In examining satisfaction with a participant’s current medication, the average rating across all participants was 4.6. When examining satisfaction by condition, participants significantly differed among each of the conditions (migraine=4.2, MS=5.1, and RA=4.4; *F*_2,1638_=62.97, *P<*.01). Similarly, when exploring perceived effectiveness with current medication, the average rating was 4.6 across all participants. A significant difference was also seen between each of the conditions in participants’ perceived effectiveness ratings (migraine=4.4, MS=5.0, and RA=4.3; *F*_2,1638_=41.23, *P<*.01). Given the parallels in the satisfaction and perceived efficacy ratings, correlation was conducted between these ratings to verify the relationship. As expected, both within and across all conditions, ratings of current medication satisfaction and current medication perceived efficacy were strongly correlated (all participants, *r*_1639_=0.90, *P<*.01; migraine, *r*_522_=0.91, *P<*.01*;* MS, *r*_615_=0.85, *P<*.01*;* RA, *r*_498_=0.93, *P<*.01)*.*

### Relationship Between Time and Treatment Satisfaction and Perceived Effectiveness

Time on treatment for a participant’s condition and time on current medication were examined to identify if these factors are related to treatment satisfaction and perceived efficacy. It was found that as the duration that the participant was taking medication for the condition increased, so did the participant’s satisfaction with and perceived efficacy of the current medication. Satisfaction was significantly correlated with time on treatment for the participant’s condition within each of the three surveyed conditions, but not across the entire sample (all participants, *r*_1639_=0.04, *P=*.09; migraine, *r*_522_=0.10, *P=*.03*;* MS, *r*_615_=0.08, *P*=.047*;* RA, *r*_498_=0.17, *P<*.01; Table 2). Similarly, perceived treatment efficacy was significantly correlated with time on treatment within each of the three surveyed conditions, as well as across the entire sample (all participants, *r_s_*_1639_*=*0.08, *P*<.01; migraine, *r_s_*_522_=0.09, *P=*.04*;* MS, *r_s_*_615_=0.11, *P*=.01*;* RA, *r_s_*_498_=0.17, *P*<.01.

**Table 2:** Medication satisfaction and perceived effectiveness by time on treatment.

Medication ratings		Migraine		MS^a^		RA^b^		All	
**Mean satisfaction ratings by time on treatment**									
	Less than 1 year		4.1		4.7		4.0		4.4
	1 to 4 years		3.9		5.2		4.2		4.6
	5 to 10 years		4.2		5.2		4.5		4.7
	More than 10 years		4.3		5.2		4.7		4.6
**Mean perceived effectiveness ratings by time on treatment**									
	Less than 1 year		4.2		4.6		4.0		4.3
	1 to 4 years		4.0		5.1		4.2		4.5
	5 to 10 years		4.4		5.2		4.5		4.7
	More than 10 years		4.5		5.2		4.8		4.7

^a^MS: multiple sclerosis.

^b^RA: rheumatoid arthritis.

Within each of the three conditions, as well as across the entire sample, time on current medication was significantly correlated with both satisfaction (all participants, *r_s_*_1639_*=*0.22, *P*<.01; migraine, *r_s_*_522_=0.24, *P*<.01*;* MS, *r_s_*_615_=0.14, *P<*.01*;* RA, *r_s_*_498_=0.29, *P*<.01) and perceived efficacy of the participant’s current medication (all participants, *r_s_*_1639_*=*0.25, *P*<.01; migraine, *r_s_*_522_=0.26, *P*<.01*;* MS, *r_s_*_615_=0.21, *P<*.01*;* RA, *r_s_*_498_=0.28, *P*<.01) (Table 3).

**Table 3:** Medication satisfaction and perceived effectiveness by time on current medication.

Medication ratings		Migraine		MS^a^		RA^b^		All	
**Mean rating of satisfaction by time on current medication**									
	Less than 3 months		3.5		4.8		3.6		3.8
	3 to 6 months		4.0		4.8		4.1		4.3
	6 months to 1 year		4.0		5.0		4.7		4.6
	1 to 2 years		4.2		5.2		4.3		4.8
	More than 2 years		4.5		5.3		4.9		4.9
**Mean rating of perceived effectiveness by time on current medication**									
	Less than 3 months		3.6		4.6		3.6		3.8
	3 to 6 months		4.1		4.6		4.1		4.3
	6 months to 1 year		4.1		4.8		4.7		4.6
	1 to 2 years		4.2		5.0		4.3		4.7
	More than 2 years		4.7		5.3		4.8		5.0

^a^MS: multiple sclerosis.

^b^RA: rheumatoid arthritis.

### Relationship Between Medication Experience and Satisfaction and Perceived Effectiveness

The median number of medications ever taken for each participant’s health condition was calculated per condition: all conditions = 4, migraine=7, MS=2, and RA=4. From these medians, participants were split into groups by number taken: Up through the median for each condition and over the median for each condition. Satisfaction and perceived effectiveness ratings were then correlated with these categories to find that there is a negative relationship with those who have tried more medications being less satisfied (Table 4). Significant negative correlations were found within migraine and MS participants, as well as across the entire sample for both satisfaction (all participants, *r_s_*_1639_*=*−0.09, *P*<.01; migraine, *r_s_*_522_=−0.12, *P*<.01*;* MS, *r_s_*_615_=−0.10, *P*=.01*;* RA, *r_s_*_498_=−0.02, *P*=.59) and perceived effectiveness (all participants, *r_s_*_1639_*=*−0.12, *P*<.01; migraine, *r_s_*_522_=−0.15, *P*<.01*;* MS, *r_s_*_615_=−0.15, *P*<.01*; r_s_*_498_=−0.03, *P*=.50).

**Table 4:** Medication satisfaction and perceived effectiveness by medication experience.

Mean rating		Migraine	MS^a^	RA^b^	All
**Mean rating of satisfaction by medication experience**					
	Up through median	4.4	5.3	4.4	4.7
	Over median	4.0	5.0	4.3	4.5
**Mean rating of perceived effectiveness by medication experience**					
	Up through median	4.6	5.2	4.4	4.8
	Over median	4.1	4.8	4.3	4.4

^a^MS: multiple sclerosis.

^b^RA: rheumatoid arthritis.

### Demographics in Relation to Medication Satisfaction and Perceived Effectiveness

In addition to exploring treatment experience and time in relation to satisfaction and perceived effectiveness, the participant’s age and frequency of seeing an HCP were also examined. As age increased, so did the participant’s satisfaction (all participants, *r_s_*_1639_*=*0.09, *P*<.01; migraine, *r_s_*_522_=0.13, *P*<.01*;* MS, *r_s_*_615_=0.04, *P*=.30*;* RA, *r_s_*_498_=−0.12, *P*<.01) and perceived effectiveness (all participants, *r_s_*_1639_*=*0.11, *P*<.01; migraine, *r_s_*_522_=0.17, *P*<.01*;* MS, *r_s_*_615_=0.07, *P*=.11*;* RA, *r_s_*_498_=0.13, *P*<.01) with the current medication. These correlations are significant for the entire sample, as well as for migraine and RA participants, but not for MS participants (Table 5).

**Table 5:** Medication satisfaction and perceived effectiveness by participant age.

Mean rating		Migraine	MS^a^	RA^b^	All
**Mean rating of satisfaction by participation age (years)**					
	<40	3.9	5.1	4.0	4.4
	40-49	4.2	5.1	4.5	4.6
	50-59	4.2	5.2	4.2	4.6
	60+	4.7	5.3	4.7	4.0
**Mean rating of perceived effectiveness by participation age (years)**					
	<40	4.1	4.8	4.0	4.3
	40-49	4.3	5.1	4.5	4.6
	50-59	4.4	5.1	4.1	4.6
	60+	4.9	5.2	4.7	4.9

^a^MS: multiple sclerosis.

^b^RA: rheumatoid arthritis.

A negative relationship was found between the frequency of HCP visits with both satisfaction and perceived effectiveness, indicating that with fewer HCP visits come higher contentment and perceived value (Table 6). A significant negative correlation was found for satisfaction within migraine and RA participants, as well as the entire sample (all participants, *r_s_*_1639_*=*−0.22, *P*<.01; migraine, *r_s_*_522_=−0.23, *P*<.01*;* MS, *r_s_*_615_=−0.08, *P*=.05*;* RA, *r_s_*_498_=−0.14, *P*<.01); perceived efficacy was significant within all conditions and across the entire sample (all participants, *r_s_*_1639_=−0.24, *P*<.01; migraine, *r_s_*_522_=−0.28, *P*<.01*;* MS, *r_s_*_615_=−0.10, *P*=.01*;* RA, *r_s_*_498_=−0.18, *P*<.01).

**Table 6:** Medication satisfaction and perceived effectiveness by frequency of health care professional visits.

Mean ratings		Migraine	MS^a^	RA^b^	All
**Mean rating of satisfaction by frequency of HCP^c^ visits**					
	Once a month or more	3.7	4.9	4.0	4.0
	Every 2-3 months	4.2	5.0	4.4	4.5
	Twice a year	4.6	5.2	4.8	5.1
	Once a year, when I relapse/need to	4.7	5.2	4.9	4.9
**Mean rating of perceived effectiveness by frequency of HCP visits**					
	Once a month or more	3.7	4.8	3.9	4.0
	Every 2-3 months	4.3	4.9	4.4	4.5
	Twice a year	4.9	5.2	4.9	5.1
	Once a year, when I relapse/need to	4.9	5.2	4.9	5.0

^a^MS: multiple sclerosis.

^b^RA: rheumatoid arthritis.

^c^HCP: health care professional.

### Determining Medication Efficacy

When asked to select from a list of factors that helped participants to determine how well their current medication is working, participants across all three conditions surveyed were most likely to select their ability to perform day-to-day activities and the number of symptoms experienced (Table 7).

**Table 7:** Top factors selected by participants for determining medication effectiveness (N=1641).

Factors for determining medication effectiveness	*X*^2^ (2)	P value	Migraine, n (%)	MS^a^, n (%)	RA^b^, n (%)	All, n (%)
My ability to perform my day-to-day activities	187.4	<.01	462 (88.2)	384 (62.2)	461 (92.2)	1307 (79.65)
The number of symptoms I experience	74.1	<.01	358 (68.3)	339 (54.9)	396 (79.2)	1093 (66.61)
The number of relapses/exacerbations I experience	46.9	<.01	319 (60.9)	393 (63.7)	222 (44.4)	934 (56.92)
My ability to sleep	118.1	<.01	221 (42.2)	112 (18.2)	232 (46.4)	565 (34.43)
My mood or level of happiness/depression	62.9	<.01	205 (39.1)	147 (23.8)	168 (33.6)	520 (31.69)
My ability to exercise	100.5	<.01	118 (22.5)	143 (23.2)	238 (47.6)	499 (30.41)

^a^MS: multiple sclerosis.

^b^RA: rheumatoid arthritis.

## Discussion

### Principal Findings

Further validating the relationship between medication satisfaction and perceived treatment effectiveness, large effect sizes (ranging from 0.85 to 0.93) were observed across conditions between ratings of medication satisfaction and perceived treatment effectiveness. Interestingly, however, mean treatment satisfaction and treatment effectiveness ratings were higher among patients with MS than patients with migraine or RA, which may be related to differences in the demographic composition of the subgroups (eg, more male patients were represented in the MS sample compared with the other groups) and/or which may reflect differences in overall condition management (eg, patients with MS report more frequent HCP visits compared with the other groups).

Across all three conditions, a time dimension—regardless of how it was measured (time since diagnosis, time since starting medication for condition, and time on current medication)—also showed a positive relationship with medication satisfaction and perceived treatment effectiveness. More experience with the condition (and with medication for the condition) may lead to differing/more realistic expectations for treatment effectiveness and/or may give patients time to find a treatment that *works*. With that said, the research also demonstrates that patients who cycle through multiple medications and/or who are in more regular contact with their HCP are more likely to report lower perceived treatment effectiveness (the former was only significant for patients with migraine and MS).

Overall, these findings suggest that clinicians’ understanding of disease history (time since diagnosis) and treatment history (time since starting on medication for condition, number of over-the-counter and prescription medications tried, and time on current medication) will help inform a perspective on patient mindset regarding treatment effectiveness. Are patients at a point where they have accepted the diagnosis, they understand expectations for treatment effectiveness, and they have found a treatment that works for them? Or, have patients cycled through multiple medications without success and made frequent visits to discuss next steps?

The research also found that the ability to perform daily activities was, by far, the most likely way in which patients with migraine or RA determined whether a treatment was working for them, whereas patients with MS were almost equally as likely to factor in both ability to perform daily activities and number of relapses/exacerbations. However, it is important that clinicians not forget to inquire about potential other factors that may have an impact on perceived treatment effectiveness. These secondary factors included number of symptoms experienced (across all three conditions), ability to sleep (for patients with migraine and RA, in particular), and ability to exercise (for patients with RA, in particular). It is also interesting to note that patients are less likely to indicate that the assessment of their HCP influenced their determination of treatment effectiveness—instead relying more on their personal experience.

Given the link between perceived treatment effectiveness and treatment adherence, an understanding of the factors that patients use to determine whether a treatment *works* can help clinicians tailor their patient interactions to more specifically discuss these factors. Questions around level of daily functioning and symptoms, as well as sleep and exercise patterns, should be used to help clinicians better understand the treatment experience. As needed, clinicians should be prepared to initiate conversations early if patients point to issues with these aspects to ensure continued adherence if appropriate. There should also be recognition that the clinician’s assessment that a treatment is working may not be mirrored by the patient, and that treatment goals may differ between the clinician and patient [20-22]. Recent views expressed by Crum and Zuckerman [23] reinforce the importance of clinician-patient conversations around perceived treatment effectiveness, including identifying the origin of a patient’s mindset around treatment and how fixed or malleable that mindset may be. Brown [24] makes a similar point regarding the importance of close and careful listening to patients, in her discussion of *What Patients Say, What Doctors Hear* by Danielle Ofri [25].

### Limitations

The study’s sample size had an adequate level of statistical power; however, interpretation of these data was limited by design issues inherent with using convenience sampling and self-report data, which is subject to recall and participation bias. Respondents represent those who are engaged with online health communities and may not represent or be generalizable to the broader patient populations of each condition. For example, more women completed the survey than men, which should be noted as a limitation. In addition, patients recruited through these methods may have had increased knowledge about the progression, treatment, and coping strategies of the disease, which may have influenced study results. More research needs to be done to determine if study findings are consistent across patient populations recruited in other ways.

Additional research into the factors underlying perceived treatment effectiveness is needed, incorporating aspects not directly assessed in this research (eg, side effect/tolerability experiences) and/or focusing more explicitly on prescription (rather than over-the-counter) treatments. This research also did not directly address the relationship between perceived treatment effectiveness and compliance, although other research has suggested a strong relationship between the two (eg, for use of mental health services) [26].

### Conclusions

Time since diagnosis, time since starting medication for condition, and time on current medication showed a positive relationship with medication satisfaction and perceived treatment effectiveness. In addition, more patient experience with the condition (and with medication for their condition) may lead to more realistic treatment expectations and/or may give patients time to find a treatment that they believe works for them. Conversely, lower perceived treatment effectiveness and multiple medication attempts appear to prompt more frequent HCP visits, likely increasing health care costs and placing potential strain on the HCP-patient relationship.

Given the link between perceived treatment effectiveness and adherence, an understanding of the factors patients used to determine whether a treatment is effective can help clinicians tailor their patient interactions to more specifically discuss these factors. Clinicians should ask more questions around symptoms and daily functioning, as well as perceived treatment effectiveness, to better understand the treatment experience. Additional research into the factors underlying perceived treatment effectiveness is needed, incorporating aspects not directly assessed in this research, such as tolerability.

## Abbreviations

HCP: health care professional
IP: Internet Protocol
MS: multiple sclerosis
RA: rheumatoid arthritis
TSQM: Treatment Satisfaction Questionnaire for Medication

## References

[1] World Health Organization (2003). Adherence to Long-term Therapies: Evidence for Action.

[2] Lavsa SM, Holzworth A, Ansani NT (2011). Selection of a validated scale for measuring medication adherence. J Am Pharm Assoc (2003).

[3] Hughes DA, Bagust A, Haycox A, Walley T (2001). The impact of non-compliance on the cost-effectiveness of pharmaceuticals: a review of the literature. Health Econ.

[4] Scarlett W, Young S (2016). Medical noncompliance: the most ignored national epidemic. J Am Osteopath Assoc.

[5] Jin J, Sklar GE, Oh VM, Li SC (2008). Factors affecting therapeutic compliance: a review from the patient's perspective. Ther Clin Risk Manag.

[6] Lam WY, Fresco P (2015). Medication adherence measures: an overview. Biomed Res Int.

[7] Svarstad BL, Shireman TI, Sweeney JK (2001). Using drug claims data to assess the relationship of medication adherence with hospitalization and costs. Psychiatr Serv.

[8] Rajpura J, Nayak R (2014). Medication adherence in a sample of elderly suffering from hypertension: evaluating the influence of illness perceptions, treatment beliefs, and illness burden. J Manag Care Pharm.

[9] Hirsh AT, Atchison JW, Berger JJ, Waxenberg LB, Lafayette-Lucey A, Bulcourf BB, Robinson ME (2005). Patient satisfaction with treatment for chronic pain: predictors and relationship to compliance. Clin J Pain.

[10] Guisasola FA, Povedano ST, Krishnarajah G, Lyu R, Mavros P, Yin D (2008). Hypoglycaemic symptoms, treatment satisfaction, adherence and their associations with glycaemic goal in patients with type 2 diabetes mellitus: findings from the Real-Life Effectiveness and Care Patterns of Diabetes Management (RECAP-DM) Study. Diabetes Obes Metab.

[11] Chrystyn H, Small M, Milligan G, Higgins V, Gil EG, Estruch J (2014). Impact of patients' satisfaction with their inhalers on treatment compliance and health status in COPD. Respir Med.

[12] Regnault A, Balp M, Kulich K, Viala-Danten M (2012). Validation of the Treatment Satisfaction Questionnaire for Medication in patients with cystic fibrosis. J Cyst Fibros.

[13] Jneid S, Jabbour H, Hajj A, Sarkis A, Licha H, Hallit S, Khabbaz LR (2018). Quality of life and its association with treatment satisfaction, adherence to medication, and trust in physician among patients with hypertension: a cross-sectional designed study. J Cardiovasc Pharmacol Ther.

[14] Aljumah K, Hassali AA, AlQhatani S (2014). Examining the relationship between adherence and satisfaction with antidepressant treatment. Neuropsychiatr Dis Treat.

[15] Bender BG, Bender SE (2005). Patient-identified barriers to asthma treatment adherence: responses to interviews, focus groups, and questionnaires. Immunol Allergy Clin North Am.

[16] Horne R, Weinman J (1999). Patients' beliefs about prescribed medicines and their role in adherence to treatment in chronic physical illness. J Psychosom Res.

[17] Atkinson MJ, Sinha A, Hass SL, Colman SS, Kumar RN, Brod M, Rowland CR (2004). Validation of a general measure of treatment satisfaction, the Treatment Satisfaction Questionnaire for Medication (TSQM), using a national panel study of chronic disease. Health Qual Life Outcomes.

[18] Vermersch P, Hobart J, Dive-Pouletty C, Bozzi S, Hass S, Coyle PK (2017). Measuring treatment satisfaction in MS: Is the Treatment Satisfaction Questionnaire for Medication fit for purpose?. Mult Scler.

[19] González CM, Carmona L, de Toro J, Batlle-Gualda E, Torralba AI, Arteaga MJ, Cea-Calvo L (2017). Perceptions of patients with rheumatic diseases on the impact on daily life and satisfaction with their medications: RHEU-LIFE, a survey to patients treated with subcutaneous biological products. Patient Prefer Adherence.

[20] Rubin DT, Dubinsky MC, Martino S, Hewett KA, Panés J (2017). Communication between physicians and patients with ulcerative colitis: reflections and insights from a qualitative study of in-office patient-physician visits. Inflamm Bowel Dis.

[21] Pascoe K, Lobosco S, Bell D, Hoskin B, Chang DJ, Pobiner B, Ramachandran S (2017). Patient- and Physician-reported Satisfaction With Systemic Lupus Erythematosus Treatment in US Clinical Practice. Clin Ther.

[22] Tintoré M, Alexander M, Costello K, Duddy M, Jones DE, Law N, O'Neill G, Uccelli A, Weissert R, Wray S (2017). The state of multiple sclerosis: current insight into the patient/health care provider relationship, treatment challenges, and satisfaction. Patient Prefer Adherence.

[23] Crum A, Zuckerman B (2017). Changing mindsets to enhance treatment effectiveness. J Am Med Assoc.

[24] Brown T (2017). The power of conversation. Am J Nurs.

[25] Ofri D (2017). What Patients Say, What Doctors Hear.

[26] Lippens T, Mackenzie CS (2011). Treatment satisfaction, perceived treatment effectiveness, and dropout among older users of mental health services. J Clin Psychol.

